# Crystallization of Bis(2-hydroxyethylene) Terephthalate as a Part of a Bottle-to-Bottle Recycling Concept for Poly(ethylene terephthalate)

**DOI:** 10.1021/acs.cgd.4c00984

**Published:** 2024-08-23

**Authors:** Guido Grause, Joseph Sutton, Andrew P. Dove, Niall A. Mitchell, Joseph Wood

**Affiliations:** † School of Chemical Engineering, University of Birmingham, Edgbaston, Birmingham B15 2TT, U.K.; ‡ School of Chemistry, University of Birmingham, Edgbaston, Birmingham B15 2TT, U.K.; § Siemens Process Systems Engineering Ltd., London W6 7HA, U.K.

## Abstract

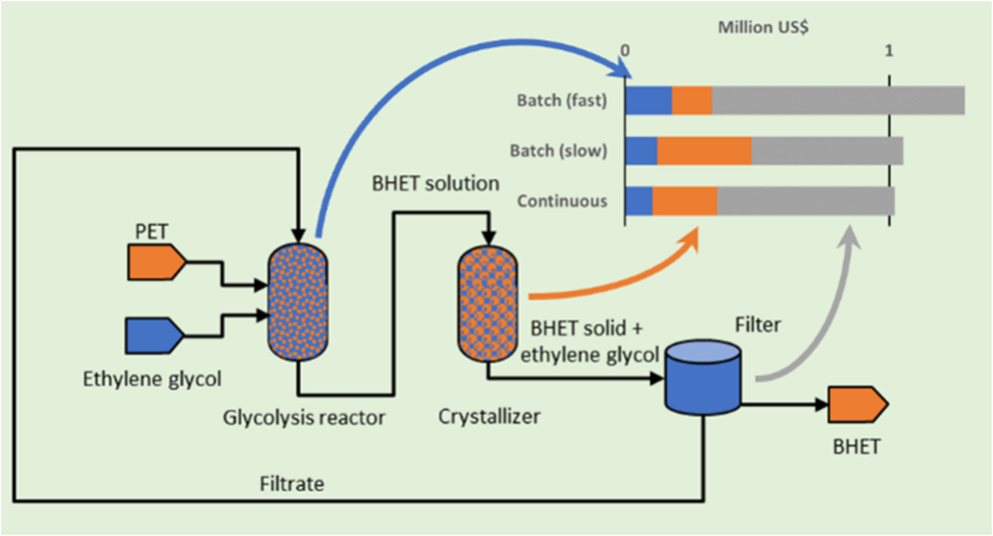

The chemical recycling of poly(ethylene terephthalate) (PET) is very attractive as PET bottle waste provides an abundant clean material with low levels of additives. One of the most promising processes is glycolysis, which depolymerizes PET in the presence of ethylene glycol. For this process, it is necessary to think through the whole concept, from the waste material to the newly polymerized virgin polymer. Most research ends with determining the yield of bis(2-hydroxyethyl)terephthalate (BHET) after glycolysis. Some research includes antisolvent crystallization with water to separate BHET from ethylene glycol. However, the subsequent separation of water and ethylene glycol is an energy-intensive process. Therefore, this work aims to directly crystallize BHET from ethylene glycol. For this reason, the crystallization of BHET was investigated experimentally. Crystallization was simulated using gPROMS Formulated Products with the aim of estimating kinetic parameters and using these to optimize an industrial process. Kinetic parameters were determined by model validation, including primary and secondary nucleation and crystal growth. The best-fitting set of kinetic parameters was used to optimize BHET crystallization in batch and continuous modes by minimizing equipment costs. Impeller parameters were found to have a great influence on crystallization performance. Ultimately, the continuous and batch processes gave comparable results in terms of equipment cost, with the batch process giving larger crystals and higher yields but the continuous process requiring a smaller crystallizer.

## Abstract 2

Cooling crystallization of bis(2-hydroxyethyl) terephthalate is studied experimentally as part of a chemical recycling process for polyethylene terephthalate plastic. The process is modeled using population balance models of gPROMS Formulated Products. The models are used to calibrate crystallization kinetics from experimental data, compare batch and continuous crystallizer configurations, and scale up and optimize the process. A cost analysis is also performed.

## 1 Introduction

Plastic pollution is one of the most pressing issues of our time.^1^ Although the use of plastics has recently been the subject of much criticism, production reached 400 million tonnes in 2022, of which 6.2 wt % was poly(ethylene terephthalate) (PET).^2^ The most common applications of PET are films, fibers, and bottles for water and soft drinks due to its high transparency and good gas barrier properties.^3^ Bottles in particular offer a high degree of recyclability if well sorted,^4^ although contamination from PET degradation and misuse must be considered.^5^

**Figure 1:**
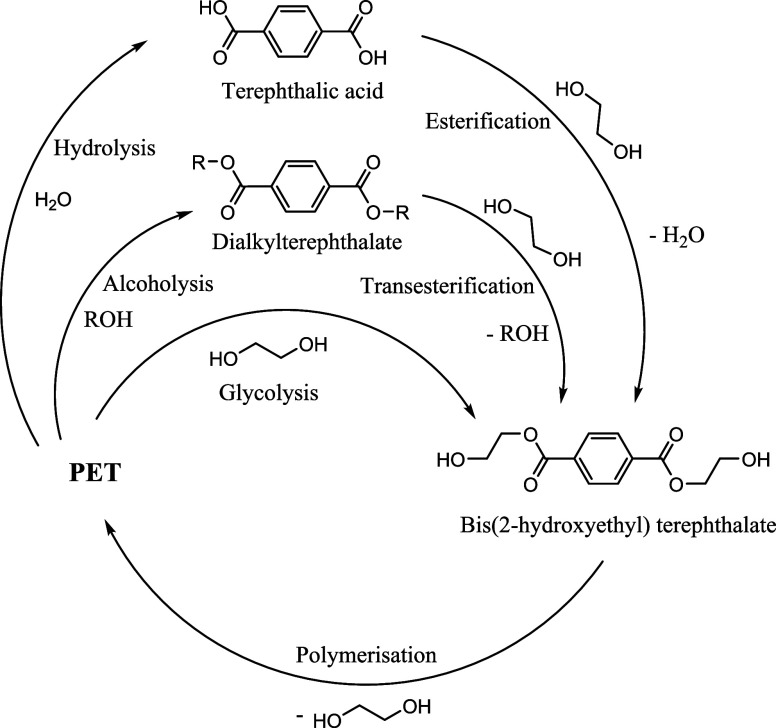
Solvolysis processes and polymerization of PET.

There are multiple techniques available for the recycling of PET. Mechanical recycling does not change the chemical composition of the polymer and involves washing, grinding, and melting the material.^6^ However, impurities within the polymer are not removed, and the reduction in molecular weight leads to a degradation of polymer properties. One way to prevent this issue is by using caustic soda to remove the top layer and subsequently elongating the polymer chains using solid-state polymerization (SSP).^7^ For a more comprehensive solution, chemical recycling can be employed, which returns PET to its monomers (Figure 1). The most extensively studied methods include hydrolysis, alcoholysis using methanol or ethanol as reactants, and glycolysis.^8^ While hydrolysis and alcoholysis provide terephthalic acid and dimethyl or diethyl terephthalate as monomers for the PET polymerization process, glycolysis provides bis(2-hydroxyethyl) terephthalate (BHET), resulting in the formation of an intermediate that can be used directly in the subsequent polymerization.

Glycolysis is typically performed below the boiling point of ethylene glycol, which is 196 °C, to avoid the need for pressurized reactors. Research has demonstrated that the reaction proceeds most effectively when the molar ratio of ethylene glycol to PET repetition unit is 15:1,^9^ resulting in a BHET mass fraction of 0.23 in the solution at the conclusion of the reaction. Nonetheless, given that this is an equilibrium reaction, oligomers—particularly the dimer—remain in the solution as a byproduct.^10^ A catalyst is necessary for the reaction. Some heterogeneous catalysts are known,^11,12^ but a homogeneous catalytic system is typically used. As PET is insoluble in ethylene glycol, the catalyst must be mobile enough to contact the PET surface. Classical metal-based catalysts like zinc or copper acetate^13^ remain under investigation. Nevertheless, metal-free alternatives are gaining popularity and becoming increasingly prevalent.^14^

The concentration of BHET after glycolysis is high enough to allow crystallization below 60 °C.^15^ Nevertheless, BHET is typically not retrieved directly from ethylene glycol, as higher yields are promised by crystallization from water, although in some instances, direct recovery from ethylene glycol by crystallization is reported.^16,17^ Duque-Ingunza et al.^18^ found that a BHET yield of 94% could be achieved by adding water to a hot solution and cooling it to 0.5 °C. Huang et al.^19^ and Goh et al.^20^ purified BHET by recrystallization from water. Goh et al.^21^ also simulated a two-stage evaporation crystallization process for ethylene glycol removal using ASPEN PLUS, which yielded 100% BHET, but information about the fate of the catalyst was not provided; crystallization with water as antisolvent gave a yield of 98%. Raheem et al.^22^ obtained comparable results in their simulation of an evaporative crystallization process. However, the subsequent separation of water and ethylene glycol is an energy-intensive process.^23^

Previous studies have mainly concentrated on BHET recovery at the end of the glycolysis process or during purification without considering crystallization kinetics. However, detailed cooling crystallization of BHET directly from ethylene glycol has not yet been reported, although this method offers a great opportunity to separate BHET from ethylene glycol without the use of any other agent. In this study, the process parameters for BHET crystallization from ethylene glycol were developed by tracking its crystallization through refractometry and simulating it using gPROMS.

gPROMS uses a one-dimensional population balance model to determine the volume-based crystal size distribution.^24^ This can be used in the first step with parameter estimation to fit experimental data and calculate the values of key parameters and in the second step to scale up processes and test different process layouts.^25^ gPROMS has recently been used to study crystallization kinetics,^26^ to control supersaturation in batch cooling processes,^24^ and to select solvents for antisolvent crystallization.^27,28^ In this study, a kinetic model was developed and validated using experimental crystallization curves, which allowed the determination of the optimal conditions for a proposed industrial process.

## 2 Experimental Section

### 2.1 Crystallization Experiment

**Figure 2:**
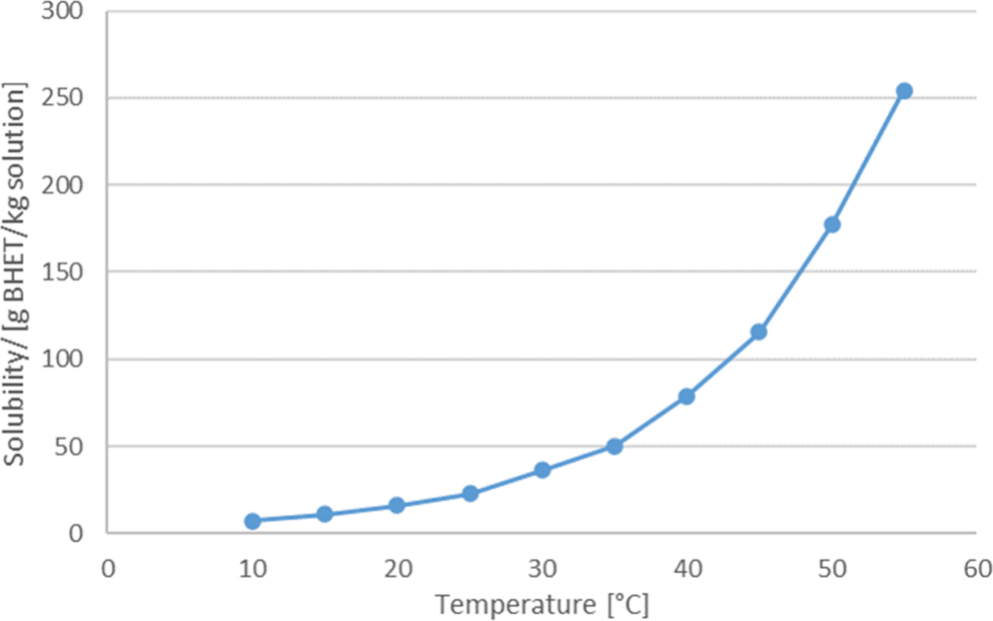
Solubility of BHET in ethylene glycol.^15^

Batch crystallization experiments were carried out using BHET and ethylene glycol (purity >99%, reagent grade) supplied by Merck. BHET was reported by the supplier to contain approximately 5 wt % oligomers. A solubility curve of BHET in ethylene glycol is shown in Figure 2 with data from Yao et al.^15^

The glass crystallizer had an internal diameter of 125 mm and a height of 185 mm. It was fitted with a 50 mm diameter propeller stirrer and a mantle heater, which was controlled by a Julabo 600F circulator. The temperature inside the crystallizer was measured using a Fisherbrand Traceable Hi-Accuracy Thermometer. The BHET concentration was assessed with a Mettler 30P refractometer between 0 and 40 °C.

For the crystallization experiments, the required amount of BHET was dissolved in 300 g of ethylene glycol at 100 °C. The clear solution was then added to 700 g of ethylene glycol at 60 °C in the crystallizer. The solution was then cooled to 45 °C and held at this temperature for 5 min in a still unsaturated state before being cooled further to 10 °C using a linear cooling profile. The oligomers crystallized between 50 and 60 °C, and the solution became turbid. The refractive index of the solution was measured every 3 min as the temperature decreased from 40 to 10 °C. For seed experiments, 8 g of BHET was dissolved in 32 g of ethylene glycol at 106 °C and then allowed to cool to room temperature, where it crystallized. The resulting suspension was refrigerated overnight and added to the crystallizer slurry between 38 and 37 °C, at which point the BHET solubility was approximately 56 g (kg solution)^−1^ (5.6 wt %). The experimental conditions are given in Table 1.

**Table 1:** Conditions for the Crystallization of BHET from Ethylene Glycol

no.	run	cooling rate [K min^–1^]	BHET concentration (without seeds) [wt %]	seed mass [g]	seed particle size *d*(0.5) [μm]
1	unseeded fast cooling	0.528	6.31	0	
2	unseeded intermediate cooling	0.301	6.52	0	
3	unseeded slow cooling	0.159	6.33	0	
4	seeded fast cooling	0.583	6.45	8.0	35.4
5	seeded intermediate cooling	0.278	6.52	8.0	22.3
6	half-seeded intermediate cooling	0.298	6.52	4.0	19.3
7	seeded slow cooling	0.155	6.33	8.0	26.8
8	unseeded high concentration	0.305	7.93	0	
9	unseeded low concentration	0.312	5.22	0	

After reaching the final temperature, a 3 mL of sample was taken from the suspension and analyzed using a Malvern Mastersizer 2000 to determine the particle size distribution. The sample was suspended in deionized water without further treatment and analyzed at an obscuration of between 10 and 20%.

To calibrate the refractometer, BHET was recrystallized to reduce the oligomer content. This was done by dissolving 90 g of BHET in 300 g of ethylene glycol at 100 °C. The clear solution was then cooled to 70 °C and held at this temperature for 30 min. The oligomers formed small crystals, which caused turbidity and could not be removed by conventional filter papers. Some of the BHET was therefore crystallized with the oligomers and filtered to remove the turbidity. The hot suspension was then filtered using preheated filtration equipment and Sartorius G393 filter paper with a diameter of 125 mm. The clear solution was then mixed with 300 mL of water, cooled to 5 °C in a refrigerator for 24 h, and filtered again. The BHET crystals were washed with 500 mL of cold water to remove residual ethylene glycol and dried at 50 °C in an oven for 3 days. The yield of dry BHET was approximately 60 g. It is not advisable to exceed the recommended drying temperature, as at higher temperatures, BHET dissolves in the residual solvent and becomes extremely hard after drying, making the product essentially insoluble in any solvent. Solutions of BHET at concentrations of 0.8, 2.0, 3.9, and 7.5 wt % were then prepared in ethylene glycol at a temperature of 60 °C and the refractive index was measured between 40 and 10 °C in 10 °C increments. At a concentration of 7.5 wt %, crystallization occurred below 10 °C.

### 2.2 Modeling

The system was modeled using gPROMS Formulated Products (version: 2022.2.0). gPROMS provides modules for simulating primary and secondary nucleation, including activated nucleation and attrition, growth and dissolution, and agglomeration.

The model is based on a one-dimensional population balance, which can be expressed as
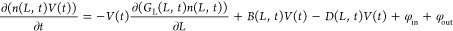
1where *n* is the particle number density, *L* is the particle length, *t* is the time, *V* is the volume of the slurry, *G* is the growth rate, and *B* and *D* are the birth and death terms, respectively. The terms φ_in_ and φ_out_ represent the flows into and out of the crystallizer, which are particularly relevant for continuous processing.^29^

It is assumed that the crystal shape remains constant throughout the process and that growth is independent of the crystal size. Nuclei are the smallest particles in gPROMS and are equivalent in size. In addition, the crystallizer is well-mixed and the phases are in thermal and mechanical equilibrium but not in chemical equilibrium. This means that particles are formed and grow from a supersaturated solution as the temperature decreases without creating a temperature gradient within the vessel.

The above model is an integral component of gPROMS. Batch and continuous crystallization can be represented, and particle size distribution evolution during the process is simulated. The lab-scale experiment was validated to obtain the kinetic data. The data were then employed to create a temperature profile for a batch process that was based on cost-effectiveness.

The viscosity and density data for ethylene glycol were provided by Engineering ToolBox.^30^ A polynomial equation can express the temperature dependence of ethylene glycol between 4.4 and 137.8 °C
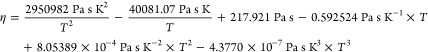
2

For the same temperature range, the density was expressed as
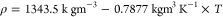
3

#### 2.2.1 Model Validation

Time, temperature, and concentration data from eight crystallization experiments (Table 1) were used for parameter estimation and one (run number 6 in Table 1) for external model validation. The kinetic parameters shown in Table 2 were chosen for parameter estimation from the experimental data. The effective diffusivity correction factor as part of the growth and dissolution kinetics was set to 1. The physical properties of the components and the transport properties of the system were calculated from the correlations given in eqs 2 and 3 or calculated using the gProms software database and were not estimated from the experimental data. Different combinations of models, including primary nucleation, activated secondary nucleation, attrition, and crystal growth, which are an inherent part of gPROMS, were previously tested to find the most appropriate model for the crystallization of BHET from ethylene glycol.

**Table 2:** Parameter Range for Initial Model Validation and Further Alterations

parameter	lower boundary	upper boundary	alteration	unit
primary nucleation				
rate constant	20	35	±1	log [m^–3^ s^–1^]
surface energy	0.003	0.007	±0.0005	J m^–2^
secondary nucleation (attrition)				
rate constant	10	50	±2	log [m^–3^ s^–1^]
size above which crystals undergo attrition	20	300	±10%	μm
kinetic order	1	3	±0.1	
growth and dissolution				
activation energy	0	30	±2	kJ mol^–1^
growth rate constant	1 × 10^–8^	0	×÷2	m s^–1^
supersaturation order	0	3	±0.1	

As a result, the most promising model consisted of primary nucleation, secondary nucleation by attrition, and Mersmann growth and dissolution kinetics. Primary nucleation^31^ was described by
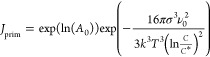
4where *J*_prim_ is the primary nucleation rate, *A*_0_ is the pre-exponential factor, σ is the surface energy, *v*_0_ is the molecular volume, *k* is the Boltzmann constant, *T* is the absolute temperature, *C* is the BHET concentration, and C* is the solubility of BHET.

Secondary nucleation by attrition through crystal-impeller collisions^32^ was defined by
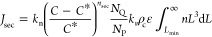
5where *J*_sec_ is the secondary nucleation rate, *k*_n_ is the secondary nucleation rate constant, *n*_sec_ is the kinetic order, *N*_Q_ is the impeller pumping number (0.79 for the impeller used), *N*_P_ is the impeller power number (1.3), *k*_v_ is the volume shape factor, ρ_c_ is the crystal density, ε is the energy dissipation rate, and *L*_min_ is the size above which crystals undergo attrition. The collision of crystals with each other was neglected due to the high viscosity of ethylene glycol.

Growth and dissolution were expressed by Mersmann et al.^33^ with mass transfer described by the Sherwood correlation^34^ of
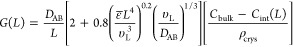
6where *G* is the linear crystal growth rate, υ is the kinematic viscosity, *C*_bulk_ is the BHET concentration in the bulk solution, and *C*_int_ is the BHET concentration at the crystal surface. The diffusion coefficient DAB is described by
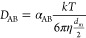
7where α is a correction factor estimated by gPROMS, η is the dynamic viscosity of ethylene glycol, and *d*_m_ is the molecular diameter of BHET.

The energy dissipation rate from eqs 4 and 5 is given by
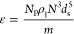
8where ρ_l_ is the density of the liquid phase, *N* is the impeller frequency, *d*_s_ is the impeller diameter, and *m* is the total mass of all liquids and solids.^29^

The surface integration, including surface diffusion of BHET toward the crystal growth site, rotation of the molecule to the correct position, and deposition on the crystal surface, is expressed by
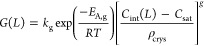
9where *k*_g_ is the growth rate constant, *E*_A,g_ is the activation energy, *g* is the order of the process, and *R* is the gas constant. Table 2 shows the parameters calculated by gPROMS.

As the range of kinetic parameters was unknown, values were randomly chosen for 8 trials within the range given in Table 2. From these trials, the best fit was selected, and its values varied, as described in Table 2, resulting in 2^8^ combinations. As a fully orthogonal design would be too much unwieldy to implement, an orthogonal array based on the Taguchi method was selected and the number of runs reduced to 12. This process was repeated until no further improvement was achieved.

#### 2.2.2 Optimization

Optimization was carried out for a batch and a continuous mixed suspension, mixed product removal (MSMPR) process. The aim was to determine whether a continuous process would be more effective for scalability and industrialization than a batch process. The optimization process requires a single parameter to be brought to a minimum or maximum. However, in this crystallization, there are three relevant parameters to consider: yield and crystal size for both processes, time for the batch process, and crystallizer volume for the continuous process. Therefore, the equipment cost, including only the cost of the glycolysis reactor, crystallizer, and filtration unit without peripherals, was introduced as the overall optimization parameter, and time, yield, and crystal size were optimized to minimize this value. Only time and volume have a direct impact on the equipment cost of the crystallizer; that is, longer crystallization times for batch processing and larger volumes for continuous crystallizers increase the equipment cost. Therefore, the time-dependent cost *C*_T_ of a batch crystallizer is calculated by
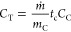
10where *ṁ* is the mass flow of the BHET solution, *m*_C_ is the capacity of the crystallizer, *t*_c_ is the crystallization time, and *C*_C_ is the cost of a batch crystallizer of defined volume, implying that the number of crystallizers required increases with crystallization time.

The volume-dependent cost *C*_V_ for one continuous crystallizer is calculated as follows
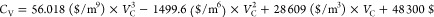
11with *V*_C_ being the volume of the crystallizer. The relationship was obtained by parameter fitting of data retrieved from Matches.^35^

For the other two parameters, other means had to be found to define their effects on the cost. Particle size becomes important when it comes to liquid–solid separation of the product.^36^ Therefore, a filtration unit was introduced, and the size of this unit was considered to define the equipment cost in relation to the crystal size *C*_CP_
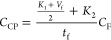
12where *K*_1_ = 938 s m^3^ and *K*_2_ = 5000 s m^–3^ are filtration constants derived from the properties of the BHET/ethylene glycol system, *V*_f_ is the filtration volume, *t*_f_ is the filtration time, and *C*_F_ is the cost of the filter unit.

For the yield, it was assumed that the filtrate was returned to the glycolysis reactor after filtration without purification (for the sake of simplicity). The noncrystallized BHET in the filtrate reduces the amount of PET that can be glycolized in the next cycle, requiring a larger reactor and crystallizer volume. The yield-related cost *C*_Y_ is then expressed as
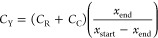
13where *C*_R_ and *C*_C_ are the costs of the glycolysis reactor and crystallizer, *x*_start_ and *x*_end_ are the mass fraction of BHET at the start and end of crystallization.

The sum of all three contributions, crystallizer, filtration unit, and glycolysis reactor, gives the total equipment cost *C*_total_ for the batch process (eq 14) and the continuous process (eq 15)
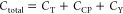
14
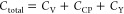
15

**Figure 3:**
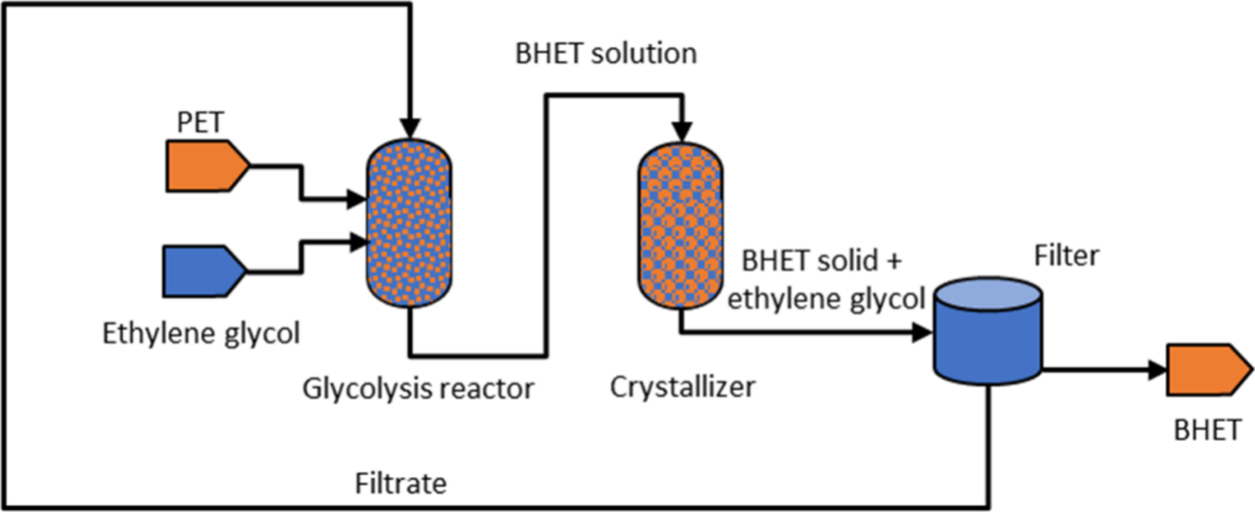
Flowchart of a 10 ktons per year industrial glycolysis process.

The optimization process was based on a pilot plant producing 10,000 tonnes of BHET per year (Figure 3). Such a plant would be capable of treating the PET bottle waste from 770,000 households in the U.K.^37^ This plant would consist of several batch reactors for glycolysis, each with a capacity of 8 m^3^ (unit cost US$134,000 each), batch crystallizers with a capacity of 8 m^3^ (US$210,200) or their continuous equivalents and drum filters with a filter area of 18.1 m^2^ (US$354,200).^35^ Residual BHET and ethylene glycol are returned to the reactor for subsequent glycolysis. A molar PET/ethylene glycol ratio of 1:15, corresponding to a BHET concentration of 23 wt % after glycolysis, and a 2 h duration for a glycolysis batch would require BHET to be crystallized from 6000 kg of solution per hour.

At the heart of this process is a crystallizer (Figure 3). Running the crystallizer at a faster rate or in a smaller volume reduces costs by reducing the number or size of crystallizers required. However, the drum filter requires a large particle size. Smaller particles reduce filtration speed and increase filter area and filtration costs. Since ethylene glycol is returned to the glycolysis reactor, a high BHET concentration reduces the amount of PET that can be processed in the next cycle. This requires a larger reactor volume and increases costs. A balance must therefore be struck between crystallization time or volume, particle size, and BHET yield.

It is expected that a solubility of BHET of 300 g (kg ethylene glycol)^−1^ (23.1 wt % BHET) at 61 °C is achieved.^15^ Eleven cooling and heating intervals were defined for batch crystallization, starting at 60 ± 5 °C and allowing cooling to a final temperature between 0 and 20 °C. For continuous crystallization, five cooling intervals were followed by 20 h at a constant temperature to achieve steady-state conditions. The viscosity of ethylene glycol increases dramatically below 0 °C, making further processing difficult. The maximum cooling and heating rates were −1 and 2 °C min^–1^, respectively. In addition, the impeller diameter and frequency were optimized based on a power number of 1.3 and a pumping number of 0.79. Lower limits were set at a diameter of 0.3 m and a frequency of 50 min^–1^. The optimization was constrained to a minimum BHET mass fraction of 2.0 wt % and a maximum particle size *D*[4,3] of 100 μm. These constraints were difficult to achieve but ensured that the optimization process attempted to achieve all of these values.

First, optimization was started using reasonable guesses for the cooling and heating intervals. The result was systematically improved by applying Taguchi designs according to the number of variables required. The designs were obtained from Minitab 21.2 and required 32 runs. The initial estimates were modified by ±20% according to the Taguchi design without violating the limits defined above. The procedure was repeated until no further improvement was achieved. The first run using the initial estimates was labeled INI, and the following 32 runs were numbered according to their appearance. The parameters from the run with the lowest cost were selected for the initial estimates of the next round and labeled INJ and INK, respectively, and the procedure was repeated.

#### 2.2.3 Sensitivity Analysis

A sensitivity analysis was performed to check the robustness of the result. This involved changing one value from the optimization at a time: each interval length, reactor volume, impeller diameter, and frequency by ±5, ±10, or ±20%; the interval length once at constant end temperature, once at constant heating/cooling rate; the initial temperature by ±0.5 °C, ±1.0 °C, or ±2.0 °C. This could improve the results compared to the original. If the cooling (−1 °C) or heating (2 °C) rate constraints were not violated, the relevant parameters were optimized iteratively.

## 3 Results and Discussion

### 3.1 Experimental Crystallization of BHET from Ethylene Glycol

The BHET used in this study was not a pure material, as it contained a certain amount of oligomers, mainly the dimer. It is probably impossible, or at least very difficult, to obtain a material free of oligomers, as monomers and oligomers coexist in equilibrium.^38^ Nevertheless, the material used is similar to what would be expected to be the product of the glycolysis of PET.

**Figure 4:**
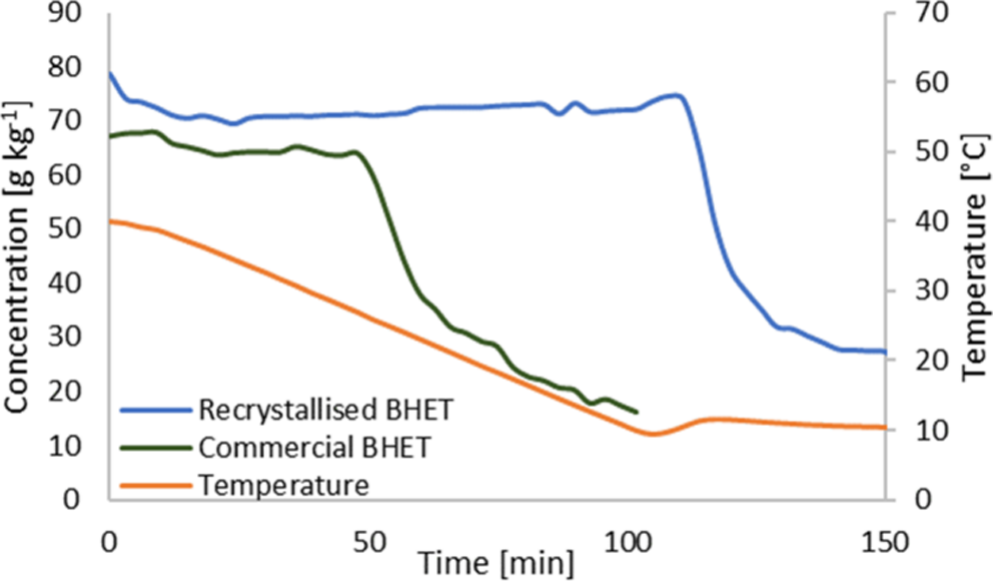
Crystallization curve of commercial and recrystallized BHET.

It is obvious that oligomers influence the crystallization process. Preliminary experiments showed that recrystallized BHET with a reduced amount of oligomers crystallized at a lower temperature than commercial BHET (Figure 4). Crystallization of recrystallized BHET started at intermediate cooling conditions below 10 °C. This was significantly lower than untreated commercial BHET, which crystallized at around 25 °C under the same conditions. In the experiments reported here, oligomers began to precipitate at around 60 °C, causing turbidity. The particle sizes of these crystals remained small, making it impossible to remove them with conventional filter papers. It is most likely that these crystals act as seeds. This could be advantageous in a practical application as they could potentially act as nucleation sites and accelerate the crystallization process.

**Figure 5:**
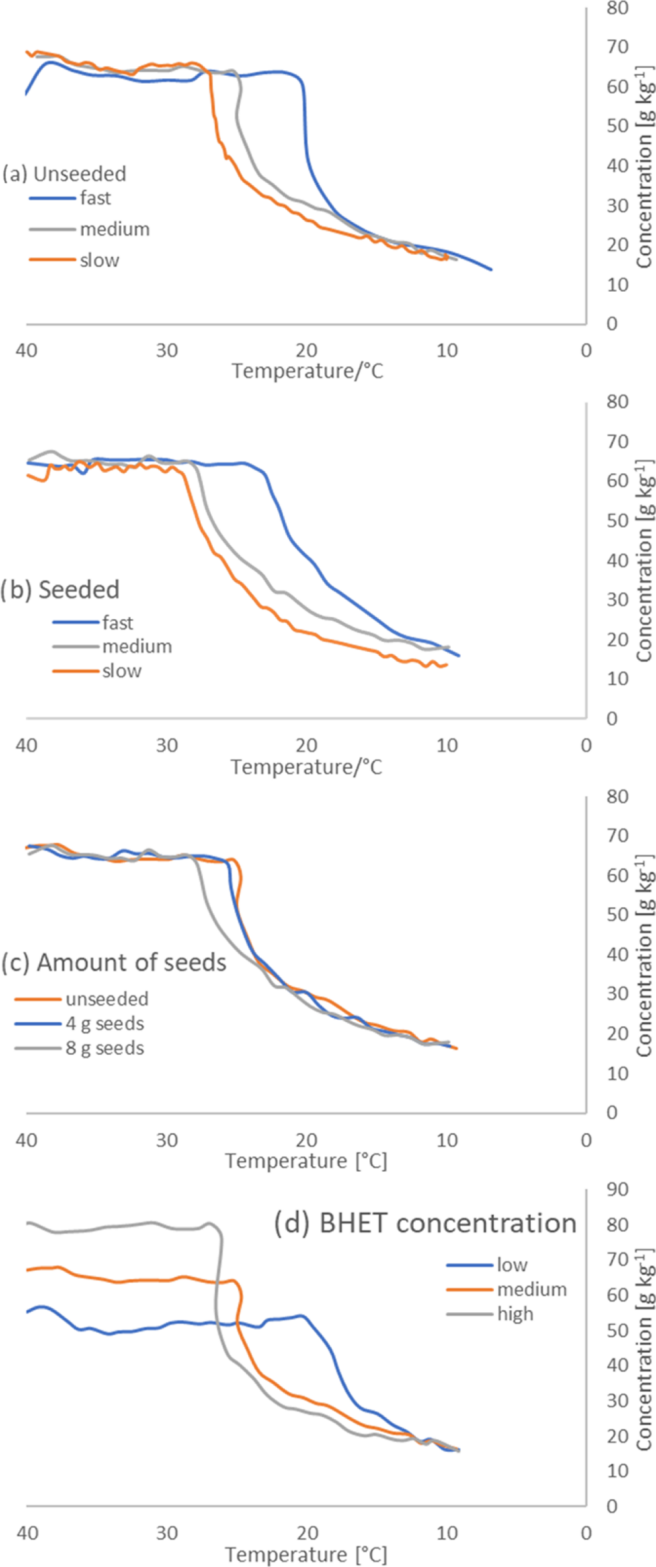
Crystallization curves of BHET from ethylene glycol: (a) unseeded, (b) seeded, (c) with different amounts of seeds, and (d) at BHET mass fractions of 5.22, 6.52, and 7.93 wt %.

Crystallization started between 27.6 °C for seeded slow cooling and 19 °C for unseeded intermediate cooling at low concentration (Figure 5). As expected, the initial crystallization temperature decreased with increasing cooling rate and decreasing BHET concentration. The crystallization of BHET was exothermic, which slowed the cooling process or even caused a slight temperature increase. Seeding had a limited effect on crystallization. The crystallization temperature increased by about 2 K with 8 g of seed. When the sample was reduced to half (4 g), the effect of seeding was barely visible (Figure 5c).

### 3.2 Model Validation

In the absence of kinetic data on the crystallization of BHET from ethylene glycol, first guesses of the kinetic parameter were randomly selected, as described in Section 2.2.1. Of the 8 runs generated in this way, one showed a promising result with a goodness of fit test χ^2^ = 233 below the χ_crit_^2^ = 269. This run was used to generate a Taguchi matrix. The best fits of these were used for another round of Taguchi matrices. This was repeated 5 times.

**Figure 6:**
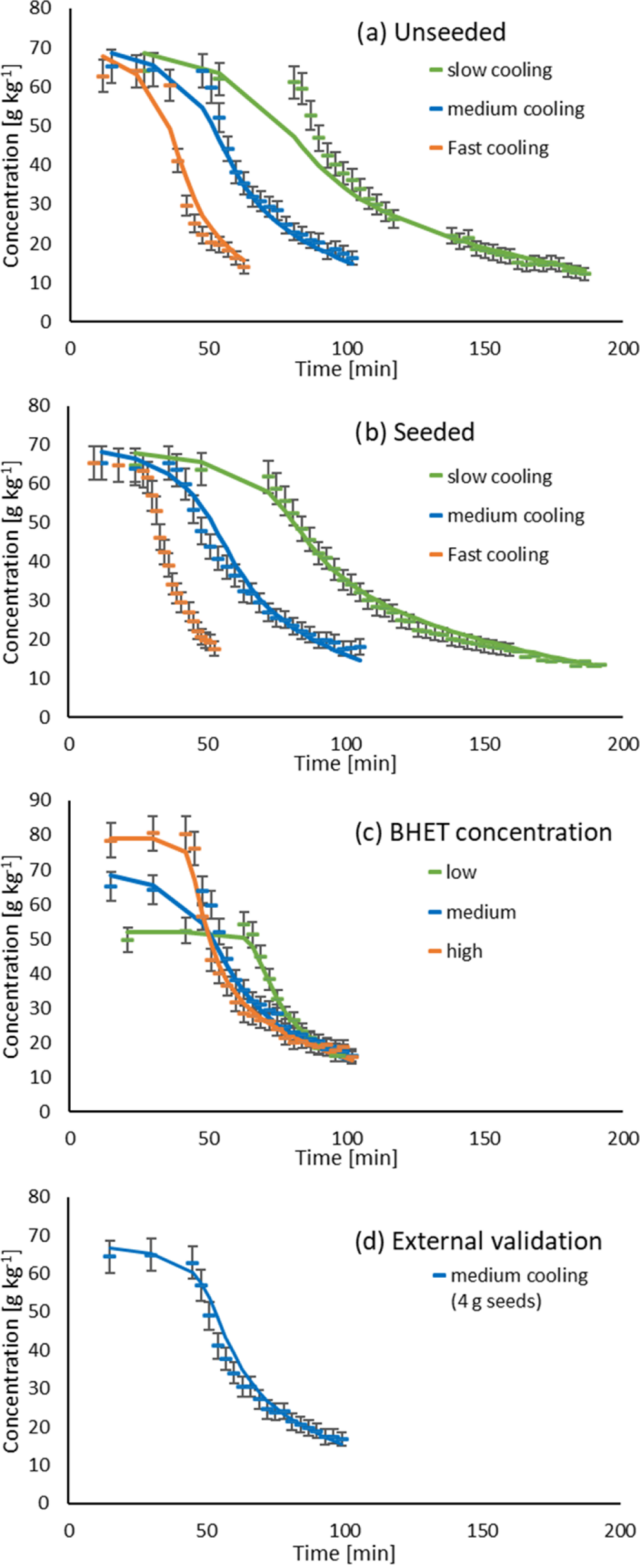
Best fit after model validation of BHET crystallization from ethylene glycol: (a) unseeded, (b) seeded, (c) at various BHET mass fractions of 5.22, 6.52, and 7.93 wt %, and (d) external validation.

**Figure 7:**
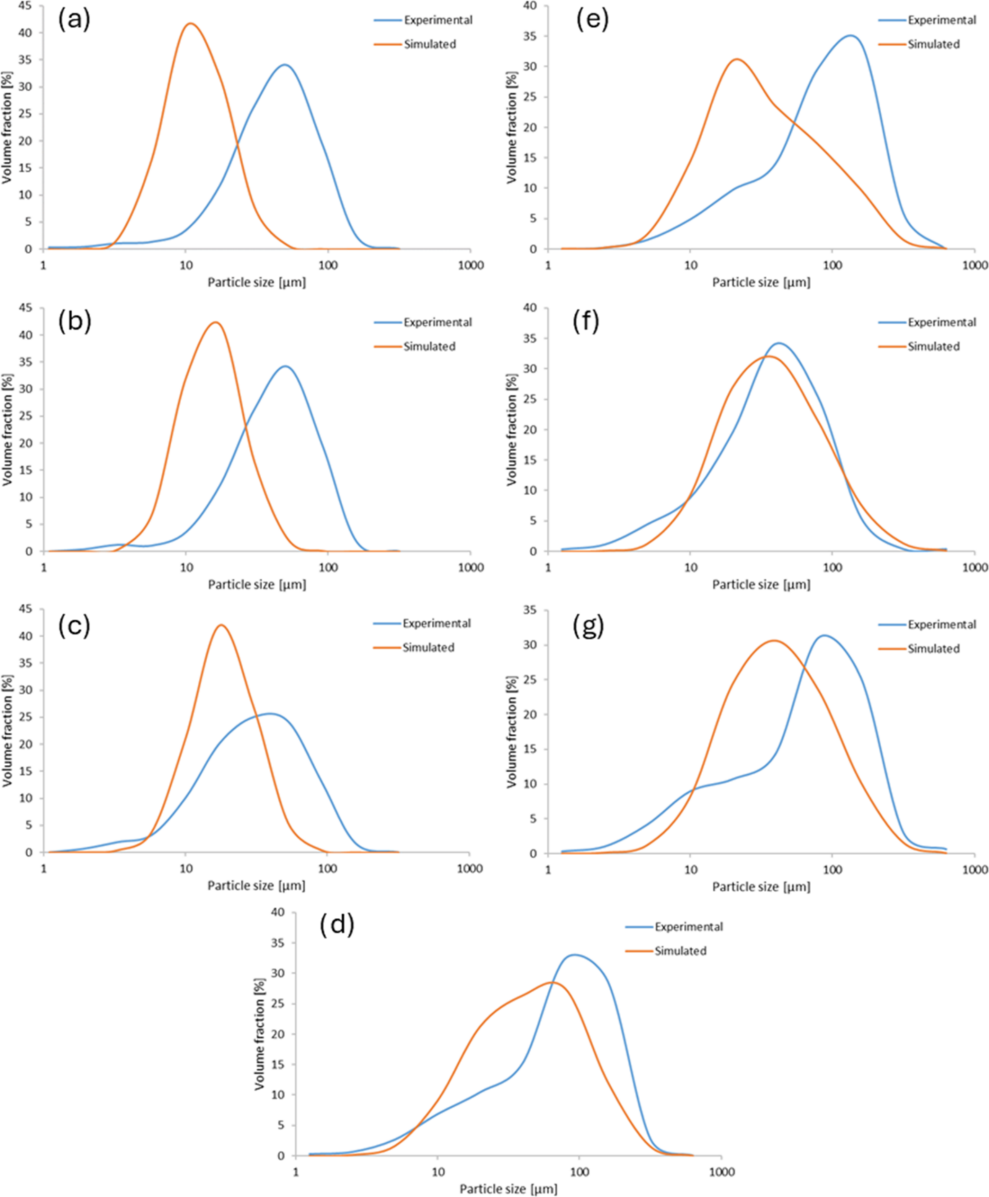
Experimental and simulated particle size distribution of (a) unseeded fast cooling, (b) unseeded intermediate cooling, (c) unseeded slow cooling, (d) half-seeded intermediate cooling, (e) seeded fast cooling, (f) seeded intermediate cooling, and (g) seeded slow cooling.

Finally, 68 sets of kinetic parameters were obtained, of which 20 had a χ^2^ below χ_crit_^2^. The best fit achieved a χ^2^ of 207 and is shown in Figure 6. Experimental data from seeded experiments, including the experimental validation run and experiments at different concentrations, were reproduced sufficiently well. The largest discrepancy between the experiment and model was observed for the unseeded slow crystallization. The simulation of the particle size distribution (PSD) also showed a better agreement with the seeded experiments (Figure 7). The bimodal nature of the PSD was correctly predicted, although the sizes of the peaks differed between the simulated and experimental curves. For the unseeded experiments, the particle size estimates were generally smaller than those derived experimentally. The model parameters for the best fit are given in Table 3.

**Table 3:** Kinetic Parameters for BHET Crystallization from Ethylene Glycol

model parameter	value	unit
primary nucleation		
rate constant	36.1719	log [m^–3^ s^–1^]
surface energy	6.64999 × 10^–3^	J m^2^
secondary nucleation (attrition)		
rate constant	33.0540	log [m^–3^ s^–1^]
size above which crystals undergo attrition	219.975	μm
order with respect to supersaturation	2.12137	
growth and dissolution		
activation energy	23.1776	kJ mol^–1^
growth rate constant	0.0359757	m s^–1^
supersaturation order	2.20127	

### 3.3 Process Optimization

**Figure 8:**
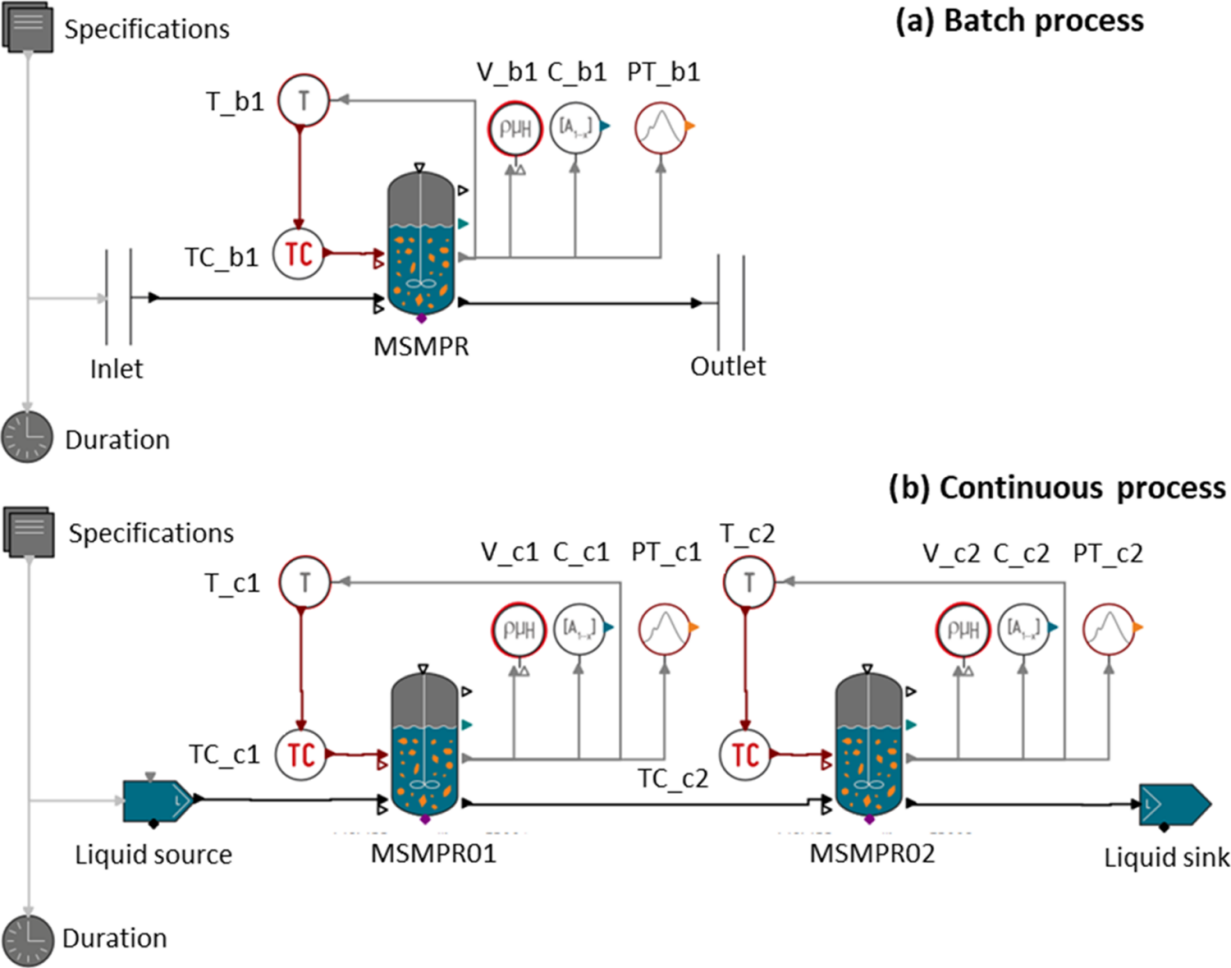
Flow sheet of (a) batch process and (b) continuous process; composition sensor (C), particle size sensor (PT), temperature sensor (T), temperature controller (TC), and viscosity sensor (V).

Optimization was carried out for a batch and a continuous process with a cascade of two crystallizers (Figure 8). For the batch process, 11 time intervals were defined with different cooling and heating rates in a temperature range between 60 and 0 °C, allowing 3 cooling and 2 heating zones. The impeller diameter and frequency were also optimized.

**Figure 9:**
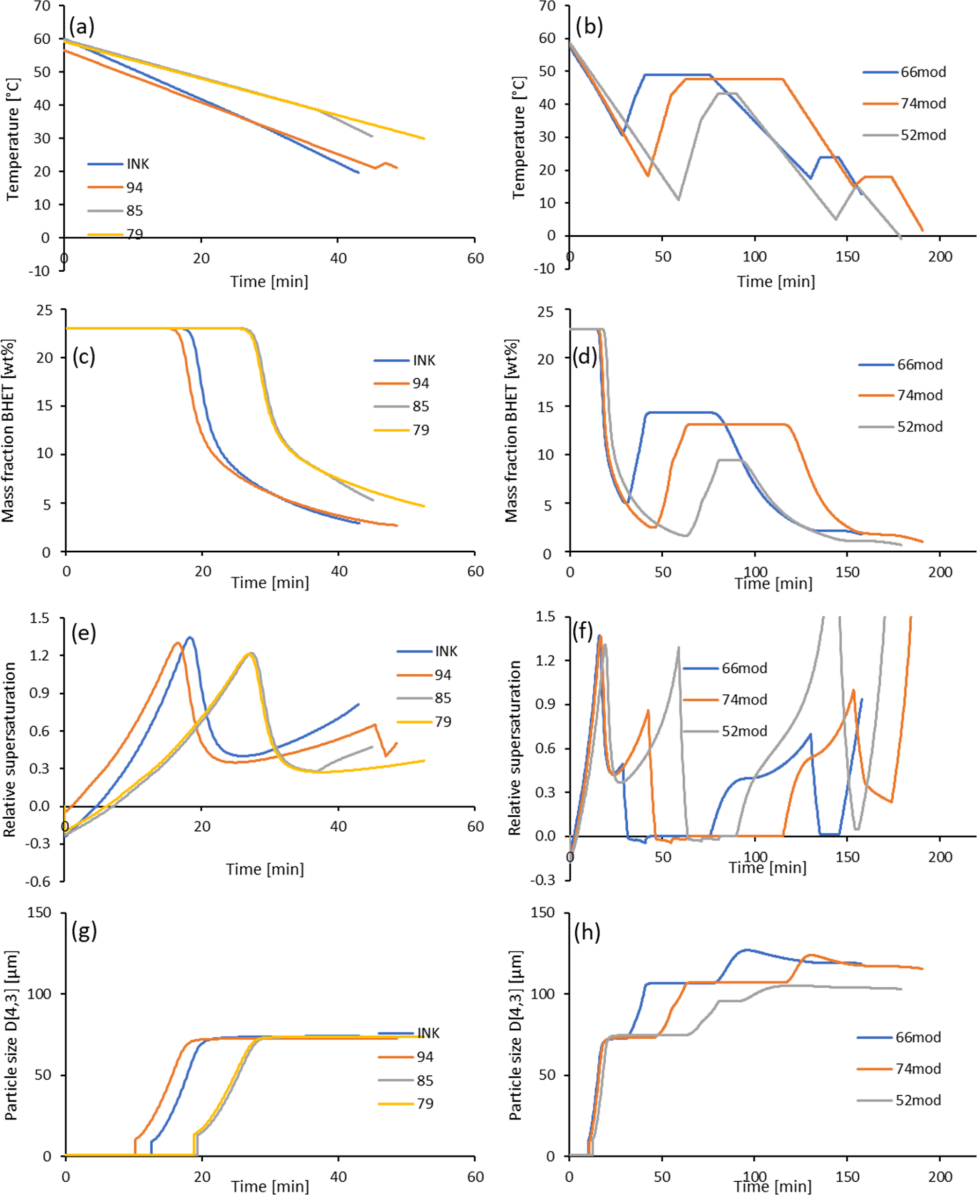
Effect of the temperature profile on the optimization result for simple temperature profiles (left) and long temperature profiles and modified impeller parameters (right): (a, b) temperature profile, (c, d) BHET mass fraction in solution, (e, f) relative supersaturation, and (g, h) particle size *D*[4,3].

As shown in Figure 9, two different strategies resulted in suitable process parameters. The runs with identifiers 79, 85, 94, and INK gave the best results in terms of equipment cost optimization (Figure 9, left, Table 4) using a simple cooling scheme at the lower limits of impeller frequency and diameter. These limits were introduced because the choice of stirring conditions was found to have a major effect on crystallization. The results suggested that the best results could be achieved in the absence of an impeller. However, this would result in very limited mass transport throughout the crystallizer. Therefore, lower limits were introduced for the impeller diameter (0.5 m) and frequency (50 min^–1^). Other runs with identifiers 52, 66, and 74 used a more complex cooling and heating scheme and higher values of the impeller parameters. Equipment costs could be reduced by setting these parameters to lower limits. Accordingly, these runs were named 52mod, 66mod, and 74mod (Figure 9, right).

**Table 4:** Optimization Results^a^

	process parameters					cost				
	particle size *d*[4,3]	mass fraction BHET	time	volume #1	volume #2	filter (particle size)	glycolysis reactor (mass fraction)	crystallizer		total
run	[μm]	[wt %]	[min]	[m^3^]	[m^3^]	[US$]	[US$]	(time) [US$]	(volume) [US$]	[US$]
batch										
66mod	119	1.79	158			753,578	93,699	556,084		1,403,361
INK	74	2.87	43			1,165,021	158,199	151,699		1,474,919
74mod	116	1.04	191			767,723	52,497	672,382		1,492,602
52mod	103	0.71	179			839,061	35,323	632,194		1,506,578
94	73	2.66	49			1,192,370	145,091	171,211		1,508,578
79	74	4.65	53			1,173,336	280,943	185,419		1,639,698
85	74	5.27	45			1,174,460	329,728	158,808		1,662,996
74	89	0.91	191			959,568	45,642	672,382		1,677,592
66	70	1.66	158			1,246,227	86,443	556,084		1,888,753
52	51	0.42	179			1,911,496	20,426	632,194		2,564,116
continuous										
INJ	89	4.16		8.8	9.5	958,941	260,314		455,151	1,674,406
53	78	2.99		8.0	9.1	1,092,801	170,790		436,960	1,700,550
50	83	4.11		10	9.1	1,022,306	260,971		466,555	1,749,832
46	66	0.42		6.9	10	1,334,027	21,275		433,151	1,788,453

a Process parameters and their associated costs are given. The extension “mod” indicates that the impeller frequency and diameter were set to lower limits of 50 min–1 and 0.5 m, respectively. This means that runs 52, 66, and 74 were carried out with a higher impeller frequency and diameter than the other runs.

Crystal size had the greatest impact on the result. A particle size *D*[4,3] of 74 μm, as observed for the INK run, resulted in a filtration cost of approximately US$1.166 × 10^6^ and a *D*[4,3] of 119 μm, as observed for the 66mod run, still yielded US$0.75 × 10^6^. This was more than half of the total cost of the process. The time required ranged from 43 min for the simple cooling profiles to 191 min for the complex ones, resulting in costs between US$0.15 × 10^6^ and US$0.67 × 10^6^. The impact of the mass fraction remaining in solution after filtration was rather small, as the solvent returned to the glycolysis reactor along with the residual BHET. The cost remained below US$100,000 as long as the BHET mass fraction did not exceed 2.0 wt % but could reach significant values above 5 wt %. The most important task was, therefore, the formation of large crystals. This could even compensate for a long crystallization time, as shown by the comparison between the 66mod and INK runs. Although 66mod required 4 times the crystallization time of INK, 66mod was the slightly cheaper process as the particle size increased from 74 to 119 μm.

With the exception of run 94, all runs of the simple cooling profile could be described by a maximum of 2 time intervals with different cooling rates (Figure 9a). Run 94 had an additional heating and cooling interval at the end of the process. This may have helped to dissolve very small crystals, although the effect is very small and not visible in the final particle size (Figure 9g). The cooling rate varied between 0.55 K min^–1^ for run 79 and 1.0 K min^–1^ for run INK and took between 43 and 52.6 min, leading to time-related costs between US$0.15 × 10^6^ and US$0.19 × 10^6^. The final temperature for runs INK and 94 reached about 20 °C, while runs 79 and 85 ended at about 30 °C. Therefore, runs INK and 94 achieved higher yields of about 88% (about US$0.15 × 10^6^ for returning BHET into the glycolysis process), while the yields for runs 79 and 85 were about 78% (about US$0.30 × 10^6^). These yields seem rather low, but it should be remembered that the filtrate was returned to the glycolysis reactor, and the dissolved BHET was not lost but enriched with newly formed BHET. Therefore, the crystallization time was a more important factor in this process than the crystallization yield. Although the cooling profiles showed some variation, the final particle size *D*[4,3] achieved was comparable for all of these runs at around 73 μm (Figure 9g).

The runs with the complex cooling profile lasted between 3 and 4 h. These produced significantly higher yields but the particle sizes were in a similar range to those above (Table 4). Although these processes were not cost-competitive, closer inspection revealed that these runs worked at higher impeller diameters and frequencies. If these parameters were set to the lower limits, particle size in particular was increased, and such processes offer an alternative to the simple cooling processes above.

These cooling profiles required between 158 min (US$0.56 × 10^6^ for time-related cost) for run 66mod and approximately 180 to 190 min (about US$0.65 × 10^6^) for runs 74mod and 52mod (Figure 9b). These were run at the lower limits for impeller diameter and frequency, although the optimization results suggested higher values for these parameters. These runs consisted of three cooling periods, interspersed with two heating periods. A heating period was usually followed by a period of constant temperature. The first cooling period was similar in time and cooling rate to the shorter runs discussed previously. This is also evidenced by the same BHET concentration, supersaturation, and particle size at the end of this period. The increasing temperature during the first heating period caused the dissolution of small crystals, the particle size increased to around 100 μm (Figure 9h), and the BHET mass fraction to 10 to 15 wt % (Figure 9d), while the relative supersaturation decreased to 0 indicating an equilibrium between crystallization and dissolution (Figure 9f). When the second cooling period started, the relative supersaturation increased again. The particle size soon reached a maximum and then stabilized at around 115 μm for runs 66mod and 74mod, indicating the formation of small crystals. During the next heating period, the relative supersaturation decreased, indicating crystal formation and growth, while the particle size remained constant. The last cooling period brought the BHET concentration to its final low level, with no change in particle size. Supersaturation remained high, indicating the possibility of even lower BHET mass fractions if more time is allowed for crystallization.

As a result, the longer crystallization time is compensated by a larger particle size and lower BHET concentration. A larger particle size allows faster filtration and reduces the equipment cost of this part of the process while the crystallization time is extended and more crystallizers of the same size are required.

Attempts with a continuous process using a single MSMPR crystallizer produced only small crystals, as expected from the complex heating profile derived for the batch process. A cascade of two crystallizers was therefore chosen as the more promising approach. For the continuous process, the crystallization time became irrelevant and was replaced by the crystallizer volume. Table 4 shows that the combined volume of the two crystallizers was around 17 m^3^ for all of the examples given. This resulted in an equipment cost of approximately US$0.45 × 10^6^ for the volume, which corresponds to a crystallization time of approximately 130 min for a batch process. As the particle size was only slightly higher and the remaining BHET mass fraction comparable to fast batch processes, the continuous processes were less efficient than the batch processes.

**Figure 10:**
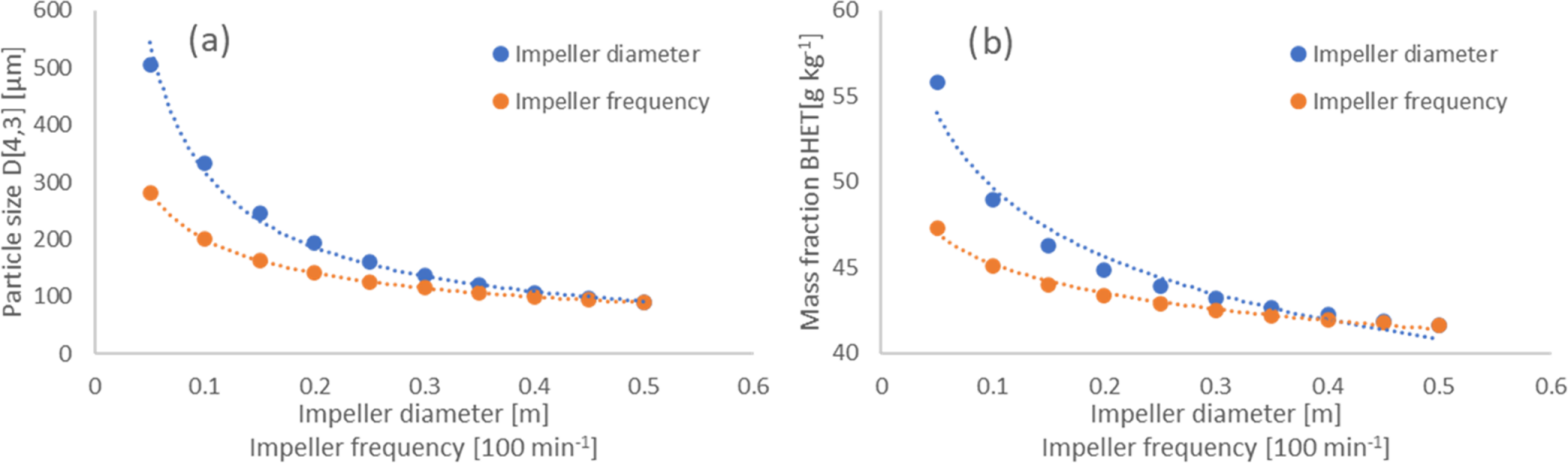
Dependence of (a) particle size and (b) BHET mass fraction on impeller diameter and frequency for run INJ in continuous mode.

As mentioned above, the impeller parameters had a strong influence on the crystallization result. It was therefore necessary to take a closer look at the crystallization behavior at different impeller settings. Figure 10 shows the dependence of particle size and BHET mass fraction on impeller diameter and frequency for the continuous run INJ at a temperature of 45.5 and 30.3 °C in the first and second crystallizers, respectively. It can be seen that the impeller diameter had a strong influence on both the final particle size and the BHET concentration. The particle size approximately doubled when the diameter was reduced by half. The BHET concentration at the end of the process increased by 20% when the diameter was reduced from 0.5 to 0.1 m. The impeller frequency had a less pronounced effect but was in the same direction on these parameters. Since contact between the impeller and the crystals caused attrition, smaller diameters and frequencies reduced the probability of contact. Crystals were allowed to grow larger. Conversely, small diameters and frequencies limit mass transport. BHET depleted near the crystal surface but remained high in the bulk solution. In other words, reducing the impeller diameter and frequency resulted in larger crystals and also in a higher residual BHET content in the solution. The next step is to determine the optimum impeller parameters for both batch and continuous processes.

**Figure 11:**
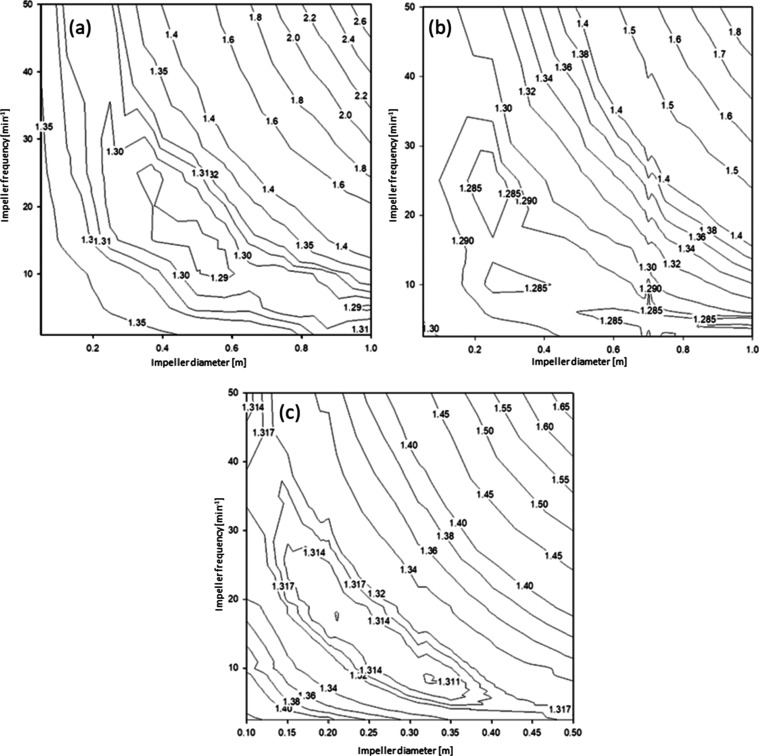
Optimization of impeller diameter and frequency for (a) run INK (fast batch process), (b) run 66 (slow batch process), and (c) run INJ (continuous process). The contour lines are labeled as millions of US$.

The strong influence of the impeller parameters on the crystallization result made it necessary to optimize both parameters. Therefore, the temperature profiles of runs INK, 66, and INJ were simulated by using different sets of impeller parameters. This allowed an optimum range to be identified, reflecting a compromise between a large crystal size and a low BHET mass fraction in the solution. Figure 11 shows that there was no single point indicating optimum conditions but rather a banana-shaped region ranging from high frequency and small diameter to low frequency and large diameter.

Equipment cost for the fast batch process INK could be as low as US$1.29 × 10^6^. This is about 13% lower than the value given in Table 4 for a 0.5 m impeller diameter and a frequency of 50 min^–1^. Values of less than US$1.3 × 10^6^ were achieved with an impeller frequency of 30 min^–1^ and a diameter of 0.25 m, down to a frequency of 5 min^–1^ at a diameter of 1.0 m. A similar behavior is observed for the slow batch process 66. The lowest value of US$1.28 × 10^6^ was slightly lower than for the INK run. In addition, the slow run was less sensitive to changes in the impeller parameters, and the applicable range was greatly extended. The fast run INK achieved a crystal size *D*[4,3] of about 89 μm (US$0.96 × 10^6^) at a BHET mass fraction of 3.17 wt % (US$0.09 × 10^6^). The slow run 66 even achieved a crystal size *D*[4,3] between 170 and 190 μm (about US$0.6 × 10^6^) at a BHET mass fraction between 2.2 and 2.5 wt % (about US$0.06 × 10^6^). The improved crystallization was achieved at the cost of an enhanced crystallization time.

The continuous run INJ did not achieve the same efficiency as the batch processes. The optimum equipment cost was about US$1.31 × 10^6^. The continuous process was also more sensitive to changes in the impeller parameters. However, the same impeller settings were used in both crystallizers of the cascade. Individual settings may give further improvement. Crystal size *D*[4,3] ranged between 250 and 390 μm (about US$0.53 × 10^6^) and BHET mass fraction between 4.6 and 5.1 wt % (about US$0.31 × 10^6^).

### 3.4 Sensitivity Analysis

The sensitivity analysis investigated the effect of changes in the process settings on the cost of the equipment. The optimization process gave an idea of how an efficient process could look like. However, it was not possible to use equipment cost directly as an objective function in the optimization. Optimization was carried out for the time, particle size, or BHET mass fraction. Therefore, apart from the fact that an optimization run with slightly different starting conditions might have led to a better result, it was always possible to achieve even better results. The aim of the sensitivity analysis is now to investigate how changes in process parameters would affect crystal size and yield. Therefore, the length and heating rate of each time interval and the starting temperature, as well as the impeller diameter and frequency and the reactor volume of the continuous crystallizer, were varied, as described in Section 2.2.3.

**Figure 12:**
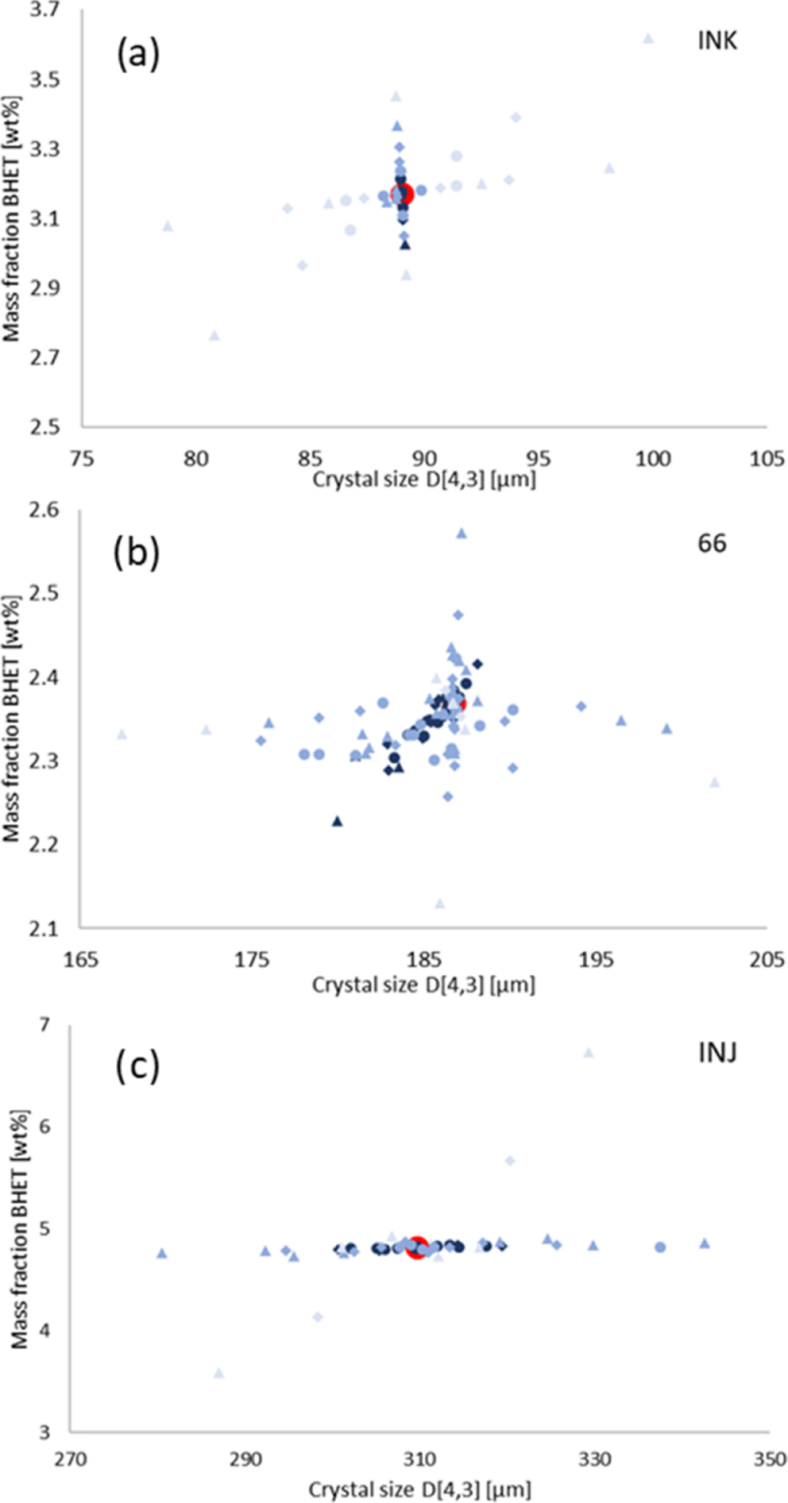
Sensitivity analysis of (a) run INK (fast batch process), (b) run 66 (slow batch process), and (c) INJ (continuous process). Particle size and BHET mass fraction are given for the initial temperature profile and modified run parameters: red circle solid Initial settings; changed settings: circle solid 5%, diamond solid 10%, triangle up solid 20%; equipment cost change: box solid <1%, blue box solid 1–2%, light blue box solid >2%.

From Figure 12a, it can be seen that the fast batch process INK was quite sensitive to some changes in the process parameters. Changing the length of the cooling time intervals had little effect on the BHET mass fraction but a significant effect on the crystal size while changing the interval end temperature changed both the crystal size and the mass fraction. In contrast, the starting temperature of the process affected only the mass fraction. A change of 5% in some parts of the cooling profile could result in additional equipment costs of up to 1.6%. Increasing the end temperature of the first cooling interval could increase the crystal size to 100 μm while increasing the BHET mass fraction to 3.6 wt %, reducing equipment costs by more than 5%. However, this would require more cooling during the second cooling interval and would violate the maximum cooling rate of 1 K min^–1^ as defined at the beginning. The impeller parameters had little effect on the result.

The sensitivity analysis for slow batch process 66 (Figure 12b) showed a more complicated pattern due to the more complex cooling profile. Most of the changes had little effect on the result, with only a few causing changes of more than 2% in the equipment cost. However, the results indicated that shortening the time of the constant temperature interval between the first heating and second cooling steps, as well as the time of the second cooling step, would not cause significant changes in crystal size or BHET mass fraction. In addition, increasing the temperature of the first heating step could improve both crystal size and BHET mass fraction. Adjusting these parameters reduced the equipment cost by approximately 18% to US$1.05 × 10^6^. The crystal size increased to 211 μm (US$0.57 × 10^6^), while the BHET mass fraction decreased slightly to 2.28 wt % (US$0.12 × 10^6^). The time was reduced by two-thirds to 101 min (US$0.36 × 10^6^). Once the optimum impeller parameters had been used, further changes in the impeller settings had little effect on the result.

Most of the changes made to the continuous process INJ (Figure 12c) had little effect on the result. Crystal size *D*[4,3] varied between 280 and 343 μm, causing only marginal changes in equipment cost between US$0.54 × 10^6^ and US$0.52 × 10^6^; BHET mass fraction changed little. The only parameter that affected equipment cost was the final temperature in the second crystallizer. Reducing this temperature resulted in smaller crystals and also a lower BHET mass fraction. The temperature was effectively reduced to 21.3 °C. Another effect seen in the sensitivity analysis was that reducing crystallizer volume had only a marginal effect on crystal size and yield. The volume could be changed to 5.36 and 1 m^3^ for the first and second crystallizers, respectively, achieving a volume-related cost of US$0.24 × 10^6^. Crystal size and yield decreased slightly but were overcompensated by the much smaller crystallizer size. In the end, the crystal size decreased to 142 μm (US$0.67 × 10^6^) and the BHET yield to 87.7% (US$0.11 × 10^6^), resulting in an equipment cost of US$1.02 × 10^6^.

Table 5 shows the final parameters for all three processes. The most efficient process was continuous crystallization, closely followed by the slow batch process. The slow batch process gave larger crystals and a higher BHET yield. This was offset by the small crystallizers required for continuous crystallization. The reader should note that although the continuous process achieved a lower yield, the cost for the yield was lower than for the slow batch process. This is due to the crystallizer volume, which reduces the result of eq 13. Although the fast batch process was the simplest, it had the highest equipment requirements. Equipment costs were more than 25% higher than those for the other two processes.

**Table 5:** Final Process Parameters^a^

	batch		
parameter	fast	slow	continuous
start temperature [°C]	62.7	57.5	
crystallizer #1			64.0
crystallizer #2			60.0
time #1 [min]	28.97	13.56	108.00
end temperature #1 [°C]	33.7	45.5	
crystallizer #1			46.7
crystallizer #2			45.5
time #2 [min]	14.05	15.00	38.40
end temperature #2 [°C]	19.6	30.5	
crystallizer #1			45.5
crystallizer #2			21.3
time #3 [min]		6.42	
end temperature #3 [°C]		41.7	
time #4 [min]		5.62	
end temperature #4 [°C]		53.0	
time #5 [min]		1.76	
end temperature #5 [°C]		52.1	
time #6 [min]		31.39	
end temperature #6 [°C]		20.7	
time #7 [min]		5.02	
end temperature #7 [°C]		23.8	
time #8 [min]		10.29	
end temperature #8 [°C]		23.8	
time #9 [min]		12.21	
end temperature #9 [°C]		12.8	
impeller diameter [m]	0.49	0.7	0.29
impeller frequency [min^–1^]	15	6	10
volume [m^3^]	8	8	
crystallizer #1			5.36
crystallizer #2			1.0
final			
crystal size *D*[4,3] [μm]	89	211	142
yield [%]	86.2	90.1	87.7
time [min]	43.0	101.3	
volume [m^3^]			6.36
equipment cost			
crystal size [US$]	957,634	574,748	674,180
yield [US$]	177,416	122,103	105,228
time [US$]	151,699	357,050	
volume [US$]			242,683
total [US$]	1,286,750	1,053,900	1,022,091

a For the continuous process, the temperatures were kept constant for 80 h after time interval #2.

## 4 Conclusions

Bis(2-hydroxyethy)terephthalate was successfully crystallized from ethylene glycol solution, and the crystallization was simulated using gPROMS Formulated Products. The crystallization was found to be adequately described by primary nucleation, attrition, and growth and dissolution kinetics. The derived kinetic data set was used to optimize crystallization in batch and continuous modes to minimize equipment costs. Crystal size, yield, and crystallization time were identified as the main factors influencing equipment cost.

Three viable solutions were identified during the optimization process: a simple batch process consisting of one cooling step, a complex batch process consisting of three cooling steps interrupted twice by heating steps, and a continuous process using a cascade of two crystallizers.

The first step was to determine the cooling profile, among other process parameters. It was found that the impeller diameter and frequency had a significant influence on the result. These were therefore optimized in the second step. As a result, all three processes showed very similar equipment costs. The sensitivity of the process parameters was then tested in the third step. While the fast batch process showed little sensitivity to changes, the slow batch process and the continuous process could be greatly reduced in time and volume, respectively. As a result, the continuous process became the most cost-effective, closely followed by the slow batch process. The slow batch process produces larger crystals and a higher yield. However, this is offset by the smaller volume of the continuous crystallizers.

## Abbreviations

*A*_0_: pre-exponential factor
*A*_t_: total crystal surface area
*B*: crystal birth term
*C*_bulk_: BHET bulk concentration
*C*_int_: BHET concentration in the liquid phase at the crystal surface
*C*_C_: batch crystallizer cost
*C*_CP_: filtration cost
*C*_F_: cost of one filtration unit
*C*_T_: time-related equipment cost
*C*_total_: total equipment cost
*C*_V_: continuous crystallizer cost
*C*_Y_: yield-related equipment cost
*c*_c_: mass density of crystals
*c**: solubility
*D*: crystal birth term
*D*[3,2]: particle size
*D*[4,3]: particle size
*D*_AB_: Diffusion coefficient of solute in the liquid phase
*d*_50_: particle size
*d*_m_: molecular diameter of BHET
*d*_S_: impeller diameter
*d*_V_: vessel internal diameter
*E*_A,g_: growth activation energy
*G*: linear crystal growth rate
*g*: supersaturation order
*J*: nucleation rate (prim: primary, sec: secondary)
*K*: solubility factor
*k*: Boltzmann’s constant
*k*_g_: growth rate constant
*k*_n_: secondary nucleation rate constant
*k*_s_: pre-exponential rate constant secondary nucleation
*k*_v_: volume shape factor
*L*: characteristic length (crystal size)
*m*: mass
*n*: crystal number density
*n*_sec_: kinetic order of the secondary nucleation
*N*: impeller frequency
*N*_P_: impeller power number
*N*_Q_: impeller pumping number
*R*: gas constant
*S*: relative saturation
*T*: temperature
*t*: time
*V*: volume
*v*_0_: molecular volume
*x*: mass fraction
α: diffusion correction factor
δ_n_: optimization parameter
ε̅: energy dissipation rate
η: dynamic viscosity
ν: number of entities
π: 3.14159
ρ: density
ρ_crys_: molar density of the crystal phase
σ: surface energy
υ: kinematic viscosity
φ: mass flow
BHET: Bis(2-hydroxyethylene)terephthalate
PET: poly(ethylene terephthalate)

## References

[ref1] Xanthos D.; Walker T. R. International policies to reduce plastic marine pollution from single-use plastics (plastic bags and microbeads): A review. Mar. Pollut. Bull. 2017, 118 (1), 17–26. 10.1016/j.marpolbul.2017.02.048. 28238328

[ref2] Plastic Europe. Plastics—the Facts 2023 2023. https://plasticseurope.org/knowledge-hub/plastics-the-fast-facts-2023/.

[ref3] Tsironi T. N.; Chatzidakis S. M.; Stoforos N. G. The future of polyethylene terephthalate bottles: Challenges and sustainability. Packag. Technol. Sci. 2022, 35 (4), 317–325. 10.1002/pts.2632.

[ref4] Welle F. Safety Evaluation of Polyethylene Terephthalate Chemical Recycling Processes. Sustainability 2021, 13 (22), 12854 10.3390/su132212854.

[ref5] Al-Sabagh A. M.; Yehia F. Z.; Eshaq G.; Rabie A. M.; ElMetwally A. E. Greener routes for recycling of polyethylene terephthalate. Egypt. J. Pet. 2016, 25 (1), 53–64. 10.1016/j.ejpe.2015.03.001.

[ref6] Suhaimi N. A. S.; Muhamad F.; Abd Razak N. A.; Zeimaran E. Recycling of polyethylene terephthalate wastes: A review of technologies, routes, and applications. Polym. Eng. Sci. 2022, 62 (8), 2355–2375. 10.1002/pen.26017.

[ref7] Cruz S. A.; Scuracchio C. H.; Fitaroni L. B.; Oliveira É. C. The use of melt rheology and solution viscometry for degradation study of post-consumer poly(ethylene terephthalate): The effects of the contaminants, reprocessing and solid state polymerization. Polym. Test. 2017, 60, 236–241. 10.1016/j.polymertesting.2017.03.026.

[ref8] Grause G. Depolymerisation of Fossil Fuel and Biomass-derived Polyesters. In Production of Biofuels and Chemicals from Sustainable Recycling of Organic Solid Waste; Fang Z.; Smith R. L. Jr; Xu L., Eds.; Springer Nature: Singapore, 2022; pp 283–316.

[ref9] Jehanno C.; Flores I.; Dove A. P.; Muller A. J.; Ruiperez F.; Sardon H. Organocatalysed depolymerisation of PET in a fully sustainable cycle using thermally stable protic ionic salt. Green Chem. 2018, 20 (6), 1205–1212. 10.1039/C7GC03396F.

[ref10] Viana M. E.; Riul A.; Carvalho G. M.; Rubira A. F.; Muniz E. C. Chemical recycling of PET by catalyzed glycolysis: Kinetics of the heterogeneous reaction. Chem. Eng. J. 2011, 173 (1), 210–219. 10.1016/j.cej.2011.07.031.

[ref11] Eshaq G.; ElMetwally A. E. (Mg–Zn)–Al layered double hydroxide as a regenerable catalyst for the catalytic glycolysis of polyethylene terephthalate. J. Mol. Liq. 2016, 214, 1–6. 10.1016/j.molliq.2015.11.049.

[ref12] Zhu M.; Li S.; Li Z.; Lu X.; Zhang S. Investigation of solid catalysts for glycolysis of polyethylene terephthalate. Chem. Eng. J. 2012, 185–186, 168–177. 10.1016/j.cej.2012.01.068.

[ref13] Al-Sabagh A. M.; Yehia F. Z.; Eissa A. M. F.; Moustafa M. E.; Eshaq G.; Rabie A. M.; ElMetwally A. E. Cu- and Zn-acetate-containing ionic liquids as catalysts for the glycolysis of poly(ethylene terephthalate). Polym. Degrad. Stab. 2014, 110, 364–377. 10.1016/j.polymdegradstab.2014.10.005.

[ref14] Kaiho S.; Hmayed A. A. R.; Chiaie K. R. D.; Worch J. C.; Dove A. P. Designing Thermally Stable Organocatalysts for Poly(ethylene terephthalate) Synthesis: Toward a One-Pot, Closed-Loop Chemical Recycling System for PET. Macromolecules 2022, 55 (23), 10628–10639. 10.1021/acs.macromol.2c01410.

[ref15] Yao H.; Yan D.; Lu X.; Zhou Q.; Bao Y.; Xu J. Solubility determination and thermodynamic modeling of bis-2-hydroxyethyl terephthalate (BHET) in different solvents. Chin. J. Chem. Eng. 2022, 45, 294–300. 10.1016/j.cjche.2021.03.024.

[ref16] Zangana K. H.; Fernandez A.; Holmes J. D. Simplified, fast, and efficient microwave assisted chemical recycling of poly (ethylene terephthalate) waste. Mater. Today Commun. 2022, 33, 104588 10.1016/j.mtcomm.2022.104588.

[ref17] Javed S.; Vogt D. Development of Eco-Friendly and Sustainable PET Glycolysis Using Sodium Alkoxides as Catalysts. ACS Sustainable Chem. Eng. 2023, 11 (31), 11541–11547. 10.1021/acssuschemeng.3c01872.

[ref18] Duque-Ingunza I.; Lopez-Fonseca R.; de Rivas B.; Gutierrez-Ortiz J. I. Process optimization for catalytic glycolysis of post-consumer PET wastes. J. Chem. Technol. Biotechnol. 2014, 89 (1), 97–103. 10.1002/jctb.4101.

[ref19] Huang J.; Yan D.; Dong H.; Li F.; Lu X.; Xin J. Removal of trace amount impurities in glycolytic monomer of polyethylene terephthalate by recrystallization. J. Environ. Chem. Eng. 2021, 9 (5), 106277 10.1016/j.jece.2021.106277.

[ref20] Goh H. W.; Salmiaton A.; Abdullah N.; Idris A. Time, Temperature and Amount of Distilled Water Effects on the Purity and Yield of Bis(2-hydroxyethyl) Terephthalate Purification System. Bull. Chem. React. Eng. Catal. 2015, 10 (2), 143–154. 10.9767/bcrec.10.2.7195.143-154.

[ref21] Goh H. W.; Salmiaton A.; Abdullah N.; Idris A. Process simulation of two-stage evaporation and crystallization systems for bis(2-hydroxyethyl) terephthalate recovery. J. Appl. Sci. 2012, 12 (15), 1547–1555. 10.3923/jas.2012.1547.1555.

[ref22] Raheem A. B.; Hassan A. B.; Noor Z. Z.; Samsudin S. B.; Hamid M. A.; Bello A.; Oladokun O.; Sabeen A. H.; Shamiri A. Process simulation of bis (2- Hydroxyethyl) terephthalate and its recovery using two-stage evaporation systems. Chem. Eng. Trans. 2018, 63, 655–660. 10.3303/CET1863110.

[ref23] Yan L.; Witt P. M.; Edgar T. F.; Baldea M. Static and Dynamic Intensification of Water–Ethylene Glycol Separation Using a Dividing Wall Column. Ind. Eng. Chem. Res. 2021, 60 (7), 3027–3037. 10.1021/acs.iecr.0c06139.

[ref24] Leeming R.; Mahmud T.; Roberts K. J.; George N.; Webb J.; Simone E.; Brown C. J. Development of a Digital Twin for the Prediction and Control of Supersaturation during Batch Cooling Crystallization. Ind. Eng. Chem. Res. 2023, 62 (28), 11067–11081. 10.1021/acs.iecr.3c00371. 37484628 PMC10360059

[ref25] Maloney A. J.; Içten E.; Capellades G.; Beaver M. G.; Zhu X.; Graham L. R.; Brown D. B.; Griffin D. J.; Sangodkar R.; Allian A.; Huggins S.; Hart R.; Rolandi P.; Walker S. D.; Braatz R. D. A Virtual Plant for Integrated Continuous Manufacturing of a Carfilzomib Drug Substance Intermediate, Part 3: Manganese-Catalyzed Asymmetric Epoxidation, Crystallization, and Filtration. Org. Process Res. Dev. 2020, 24 (10), 1891–1908. 10.1021/acs.oprd.0c00189.

[ref26] Arruda R. J.; Cally P. A. J.; Wylie A.; Shah N.; Joel I.; Leff Z. A.; Clark A.; Fountain G.; Neves L.; Kratz J.; Thorat A. A.; Marziano I.; Rose P. R.; Girard K. P.; Capellades G. Automated and Material-Sparing Workflow for the Measurement of Crystal Nucleation and Growth Kinetics. Cryst. Growth Des. 2023, 23 (5), 3845–3861. 10.1021/acs.cgd.3c00252.

[ref27] Watson O. L.; Jonuzaj S.; McGinty J.; Sefcik J.; Galindo A.; Jackson G.; Adjiman C. S. Computer Aided Design of Solvent Blends for Hybrid Cooling and Antisolvent Crystallization of Active Pharmaceutical Ingredients. Org. Process Res. Dev. 2021, 25 (5), 1123–1142. 10.1021/acs.oprd.0c00516. 34295139 PMC8289336

[ref28] Watson O. L.; Galindo A.; Jackson G.; Adjiman C. S. Computer-aided Design of Solvent Blends for the Cooling and Anti-solvent Crystallisation of Ibuprofen. In Computer Aided Chemical Engineering; Kiss A. A.; Zondervan E.; Lakerveld R.; Özkan L., Eds.; Elsevier, 2019; Vol. 46 , pp 949–954.

[ref29] Rosenbaum T.; Tan L.; Dummeldinger M.; Mitchell N.; Engstrom J. Population Balance Modeling To Predict Particle Size Distribution upon Scale-Up of a Combined Antisolvent and Cooling Crystallization of an Active Pharmaceutical Ingredient. Org. Process Res. Dev. 2019, 23 (12), 2666–2677. 10.1021/acs.oprd.9b00348.

[ref30] The Engineering ToolBox: Ethylene Glycol Heat-Transfer Fluid Properties. https://www.engineeringtoolbox.com/ethylene-glycol-d_146.html.

[ref31] Mullin J. W. Crystallization, 4th ed.; Butterworth Heinemann: Oxford, 2001.

[ref32] Evans T. W.; Sarofim A. F.; Margolis G. Models of secondary nucleation attributable to crystal-crystallizer and crystal-crystal collisions. AIChE J. 1974, 20 (5), 959–966. 10.1002/aic.690200517.

[ref33] Mersmann A.; Braun B.; Löffelmann M. Prediction of crystallization coefficients of the population balance. Chem. Eng. Sci. 2002, 57 (20), 4267–4275. 10.1016/S0009-2509(02)00343-3.

[ref34] Garside J.; Mersmann A.; Nývlt J. Measurement of Crystal Growth and Nucleation Rates, 2nd ed.; IChemE: Rugby (UK), 2002; p 202.

[ref35] Milligan D.; Milligan J. Matches. https://matche.com/equipcost/Default.html. (August 09, 2024).

[ref36] Wakeman R. The influence of particle properties on filtration. Sep. Purif. Technol. 2007, 58 (2), 234–241. 10.1016/j.seppur.2007.03.018.

[ref37] Plastor UK Plastic Recycling Statistics https://www.plastor.co.uk/statistics/plastic-recycling-uk/#:~:text=Each%20household%20generates%2013.65%20kg%20of%20plastic%20bottle,of%20those%20plastic%20bottles%20were%20sent%20for%20recyclinghttps://www.plastor.co.uk/statistics/plastic-recycling-uk/#:~:text=Each%20household%20generates%2013.65%20kg%20of%20plastic%20bottle,of%20those%20plastic%20bottles%20were%20sent%20for%20recycling. (September 28, 2024).

[ref38] López-Fonseca R.; Duque-Ingunza I.; de Rivas B.; Flores-Giraldo L.; Gutierrez-Ortiz J. I. Kinetics of catalytic glycolysis of PET wastes with sodium carbonate. Chem. Eng. J. 2011, 168 (1), 312–320. 10.1016/j.cej.2011.01.031.

